# Evaluation of a Technology-Based Peer-Support Intervention Program for Preventing Postnatal Depression (Part 2): Qualitative Study

**DOI:** 10.2196/12915

**Published:** 2019-08-29

**Authors:** Shefaly Shorey, Esperanza Debby Ng

**Affiliations:** 1 Alice Lee Centre for Nursing Studies Yong Loo Lin School of Medicine National University of Singapore Singapore Singapore

**Keywords:** depression, mothers, postpartum, qualitative, social support, telecommunication, digital health, peer support, peer-to-peer support, online support groups, internet

## Abstract

**Background:**

Social support is known to reduce risks of postnatal depression (PND) and improve maternal emotional well-being. However, the Asian cultural context is often neglected when appraising maternal needs and mothers’ preferences for social support. While many preventive efforts have experimented with technology, professionals, and paraprofessionals in providing social support to mothers in need, most studies determined the effectiveness of their interventions through quantitative measurements of maternal outcomes. Experiences and feedback from both participants and administrators are rarely discussed, especially in an Asian setting.

**Objective:**

The goal of the research was to evaluate the postnatal experiences of Asian mothers at risk of PND and the perceptions of peer volunteers regarding a technology-based peer-support intervention program (PIP).

**Methods:**

A qualitative semistructured interview was conducted with 20 Asian mothers at risk of depression (10 from the control group and 10 from the intervention group) and 19 peer volunteers from a randomized controlled trial. The PIP included weekly correspondence between peer volunteers and mothers through any telecommunication means over 4 weeks. All interviews were approximately 30 to 60 minutes long, audiotaped, transcribed verbatim, and analyzed using thematic analysis. Study findings were reported according to the Consolidated Standards of Reporting Trials checklist.

**Results:**

Two overarching themes comprising five subthemes were generated: postnatal experience (a bouncy ride, a way forward) and evaluation of the PIP (valuable, flexible, and supportive program; building blocks of a good relationship; and lessons learned and the road ahead). Mothers from both the control and interventions groups were generally satisfied with hospital care and the support received from family. They also shared similar breastfeeding challenges and needs for more informed decisions and follow-up support from the hospital. However, mothers who received the PIP tended to have more positive outlooks of their birth experiences. Overall, peer volunteers and mothers involved in the PIP found the PIP useful and expressed satisfaction with the program’s flexibility. They also shared their personal takeaways, the qualities of their friendships, and the need for extended correspondence time and recommended outreach to non–at-risk mothers.

**Conclusions:**

The positive endorsement of the PIP by peer volunteers and mothers suggests the success of the PIP in maintaining positive maternal emotional well-being during the postpartum period. With the help of technology, hospitals can easily provide additional peer support to at-risk mothers in addition to existing standard care offered to these mothers.

**Trial Registration:**

ISRCTN Registry ISRCTN14864807; http://www.isrctn.com/ISRCTN14864807

**International Registered Report Identifier (IRRID):**

RR2-10.2196/resprot.9416

## Introduction

### Background on Postnatal Depression

As one of the leading causes of maternal morbidity and a potential contributor to maternal mortality, postnatal depression (PND) has garnered much attention from researchers studying women’s health over the years [1]. Apart from affecting the psychological well-being of mothers [2-4], maternal PND can potentially increase risks of depression in their partners [5] and severely hinder their children’s neurocognitive, psychological, and social development [6-8]. In consideration of long-term adverse societal consequences, various preventive measures have been implemented to battle PND, but targeting the root cause remains a challenge due to its complex multifactorial etiology (ie, biological, psychological, and social). The contribution of psychosocial factors to PND outweighs that of biological factors, with chronic social adversity in women playing a substantial role [9,10]. This is especially so in Asian countries, where polygamous marriages, conflicting influences of culture, and conflict between mothers- and daughters-in-law are unique risk factors of PND [11-13]. According to a review by Evagorou and colleagues [14], Asian and Western mothers share similar PND risk factors and have similar manifestations of physical symptoms. Differences lie in prevalence rates and cultural factors (beliefs, values, and environment). A local study has revealed a prevalence of 6.8% for PND in Singapore, which is much lower than the global average of 16% [15]. This corresponds with findings from another review [16], but the prevalence of PND varies across Asia itself as well, and South Asia collectively has higher PND rates than Western countries [17-18]. These reviews [16-18] have also highlighted cultural differences that contributed to differences in regional prevalence rates, such as Asian’s higher emphasis on family and social relationships, hence mothers’ higher dependency on their partners, their own mothers, and extended family. The lack of female empowerment, the Asian view of women as the weaker sex, and other traditional rituals such as practicing the confinement period are additional stressors for Asian mothers. Due to the collective nature of Asian societies, social support from mothers’ family members and partners plays a crucial role in mitigating PND. Therefore, positive social relationships and increased social support are exceptionally important in ensuring the healthy psychological well-being of Asian mothers [18].

### Types of and Need for Social Support

Defined as an interpersonal process whereby the provider communicates to the recipient that he or she is cared for in this reciprocal relationship [19], social support is multidimensional and varies in terms of range of support network, type, source, and quality [20]. Types of support typically include (1) emotional support, involving expressions of trust, care, and empathy; (2) instrumental support, involving tangible aid; (3) informational support, such as advice, suggestions, and information; and (4) appraisal support, including advice that allows self-evaluation [21]. Additionally, preferred types of support are largely influenced by cultural differences. In an individualistic Western culture, direct forms of support involving verbal and emotional expressions are often sought explicitly from others, whereas in a collectivistic Asian culture, indirect support through companionship and attentiveness from others is preferred [22]. Given that the impact of social support on health and recovery highly depends on the match between support provided and needed, it is necessary to consider cultural contexts when providing postpartum support to mothers in need [23].

Social support from family and partners is known to be an effective stress buffer that reduces risks of maternal PND and maintains quality of life of mothers in the postpartum period [19]. The practice of a 1-month confinement period in the Asian culture also provides most mothers with additional instrumental support in terms of infant care and household chores from confinement nannies or in-laws [24,25]. However, mothers are often dissatisfied with social support, especially due to the lack of emotional support received [24,26,27]. This can be attributed to the conservative nature of Asian societies in which direct emotional expressions are often discouraged [28,29] and the availability of emotion-focused support is rare, even from those close [22]. This highlights an unspoken need for more emotional support for Asian mothers. Mothers often mentioned a need for a close, nonjudgmental confidante to initiate support and empathize with them [4,30]. Hence, peer support is a viable option as it creates a sense of belonging, boosts parenting confidence and self-esteem among mothers, and is effective in preventing PND [3,31]. Moreover, peer support provided by peer volunteers is found to ensure continuity of support during the postpartum period and fill in for health care professionals in between clinical follow-ups [32]. Although most mothers still prioritize the need for informational support from health care professionals [33], Sjoberg and colleagues [34] revealed that new-generation mothers preferred online peer support over face-to-face or online consultations with health care professionals. Using technology to support mothers will not only increase the accessibility, availability, and affordability of maternal health care but will also remove help-seeking barriers in conservative cultures [35-37]. Additionally, Singapore has been undergoing the Smart Nation initiative since 2014 [38], which encourages health care sectors to shift to telehealth in order to optimize resources and overcome manpower constraints. Previous local studies have also seen successful results with supportive technology-based interventions involving the use of online forums and mobile apps [39,40]. Therefore, technology-based supportive interventions in this context are not considered foreign and may be more ideally suited to the needs of new-generation mothers.

### Review of Current Literature

While there are a substantial amount of randomized controlled trials (RCTs) that adopted a technology-based supportive approach, the effectiveness of these RCTs is usually determined by a quantitative analysis of maternal outcomes [41-45]. In-depth qualitative analyses of user experiences and feedback are limited. Technology-based supportive interventions are effective in reducing PND in women, and study designs primarily involved weekly telephone calls or video calls by paraprofessionals or peer volunteers [41-43]. A qualitative analysis provides a descriptive and exploratory insight of user needs that can inform the further refinement and tailoring process of an intervention program to better meet user needs. The limited number of qualitative studies and the heterogeneity of available studies in terms of support source, delivery method, and frequency warrant a need to perform a qualitative study on a technology-based peer support program in an Asian setting [46-50].

Therefore, this study aimed to evaluate the postnatal experiences of mothers at risk of PND and their perceptions of a technology-based peer support intervention program (PIP). Feedback from peer volunteers was also examined in order to gain a well-rounded perspective from both recipients and administrators.

## Methods

### Design and Setting

This descriptive qualitative study is a follow-up of an RCT [51] that examined the effectiveness of a technology-based PIP on maternal outcomes including PND, postnatal anxiety, loneliness, and perceived social support received. The original study was conducted at a tertiary hospital in Singapore where 136 mothers at risk of PND were recruited through purposive sampling and randomized into either the intervention (n=69) or control (n=69) group. Mothers at risk were identified through an initial screening using the Edinburgh Postnatal Depression Scale. Only those who scored 9 and above were recruited for the RCT. Twenty peer volunteers, mothers who had recovered from PND, were recruited through a blasting of emails to the study venue’s working community and by word of mouth to facilitate the support program. Peer volunteers then underwent a training session by a psychiatrist to learn required skills (via role play and discussions) for successful administration of technology-based peer support, which covered topics on alleviating depression, anxiety, loneliness, and how to conduct appropriate referrals to a health care professional when necessary. Details on peer volunteer training can be found in the published protocol [52]. Each peer volunteer was matched with at least three mothers. The fidelity of the PIP intervention was maintained using the strategies proposed by Bellg et al [53] and Eaton et al [54]: (1) the protocol for intervention was developed and has been published [52]; (2) to confirm dosage of the intervention, log sheets were maintained on the time and duration of contacts made with peer volunteers; (3) to ensure consistency, peer volunteers were trained by the same psychiatrist; and (4) a peer volunteer manual was developed for a standardized delivery of information.

Participants in the control and intervention groups received standard hospital postnatal care such as lactation support, parent craft teaching, and postpartum follow-up appointments with an obstetrician. In addition, participants in the intervention group had support from peer volunteers for at least 1 month postpartum involving a minimum of once-a-week correspondence between the mothers and the volunteers through any technology-based means (ie, phone calls, text messages, and WhatsApp). Frequency and duration were tailored to maternal needs. Further details have been published elsewhere [52]. The experiences of mothers in the control group were included in this follow-up to provide different perspectives and form a basis of comparison of needs and received support during the perinatal period. Prior to this, all participants were informed of this optional extended qualitative component of the study. Qualitative feedback from peer volunteers on delivery of the intervention was made mandatory upon the initial consent for the original study.

### Recruitment

Purposive sampling was used to achieve an equal number of participants from the control and intervention groups. Recruitment was conducted at 1-month postpartum through the blasting of emails to the 136 participants in the original study until data saturation was reached. Thirty-six mothers volunteered for the interviews. However, data saturation was reached at the eighth participant for both groups when no new findings emerged. Two additional mothers from each group were interviewed to confirm the findings, resulting in a total of 20 interviewed mothers (10 from the control group and 10 from the intervention group). The remaining 16 mothers consented to be excluded from the interviews. One peer volunteer had withdrawn from the original study due to time commitment issues, leaving nineteen peer volunteers in this study. Mothers and peer volunteers were informed of the approximate duration of each interview (30 to 60 minutes) and that all interviews would be audio-recorded for research purposes only.

### Data Collection

When participants were between 4 and 12 weeks postpartum, a female research assistant trained in qualitative interviewing techniques conducted individual face-to-face interviews at a time and location to each mother’s convenience, typically at the mother’s home. Participants were given pseudonyms during the interviews to protect their actual identities. Peer volunteers were only interviewed after the conclusion of the intervention for all 69 mothers, and the Peer Volunteer Activity Logs, which were used by peer volunteers to record the frequency, duration, and key points of the correspondences, were submitted to the research assistant during the interviews. Three versions of a semistructured interview guide specifically tailored to the control group, the intervention group, and peer volunteers were developed and piloted to attain a comprehensive view of the delivery and recipient of the PIP and the postnatal experiences of mothers (Multimedia Appendix 1). The interviews lasted an average 40 minutes and were subsequently transcribed verbatim. Field notes were taken during the interviews to note nonverbal cues that were used to supplement the transcripts.

### Data Analysis

A thematic analysis was conducted according to the six phases of analysis described in the research of Braun and Clark [55]. The authors read the 39 transcribed interviews multiple times to gain familiarity with the data and subsequently adopted a manual color-coding method to highlight different concepts and generate the initial codes independently. These excerpts were then extracted and put into a tabular format, entailing themes and subthemes within a new document. Data source triangulation from all three groups of participants (intervention and control groups and peer volunteers) was performed to look for common themes [56]. The themes were reviewed comprehensively for homogeneity by both authors before overarching themes were decided. To achieve confirmability and objectivity in the analysis of data, the various themes and subthemes were discussed extensively in several meetings between the two authors. Any discrepancies were discussed and clarified between the two authors until a consensus was achieved. Field notes were also constantly referred to as supplementary materials, and constant comparative analyses were performed. Themes constituting frequently reported overlapping data were selected from the authors’ independent analyses, renamed, and included in the final analysis.

### Ethical Considerations

Ethics approval was obtained from the National Health Group Domain Specific Review Board (reference number: NHG DSRB 2017/00185) of the participating hospital. Prior to obtaining the participants’ written informed consents, the participants were briefed thoroughly on the purpose of the research and were informed of their rights to withdraw at any time during the study. Study participation was strictly voluntary, and confidentiality was adhered to.

## Results

### Overview

Study findings were reported according to the Consolidated Criteria for Reporting Qualitative Research checklist (COREQ, Multimedia Appendix 2) [57]. According to our RCT results [51], the PIP was successful in significantly reducing PND symptoms at the end of three months postpartum. Although not statistically significant, there were also observable decreases in postnatal anxiety and loneliness and an increase in perceived social support at the end of three months.

### Participant Characteristics

A total of 39 interviews were completed with 20 Asian mothers (10 from the control group and 10 from the intervention group) and 19 peer volunteers. Mothers had an age range of 25 to 40 years while peer volunteers had an age range of 25 to 54 years. The majority of the mothers were Chinese (n=10), followed by Malay (n=9) and Indian (n=1), which was likewise for the peer volunteers (Chinese, n=17; Malay, n=1; Indian, n=1). All 10 of the mother-volunteer dyads corresponded (regarding emotional needs and available resources) through WhatsApp messages at least once a week; only five dyads attempted to schedule a weekly phone call. Further details of the participants and peer volunteers can be found in Table 1.

**Figure 1:**
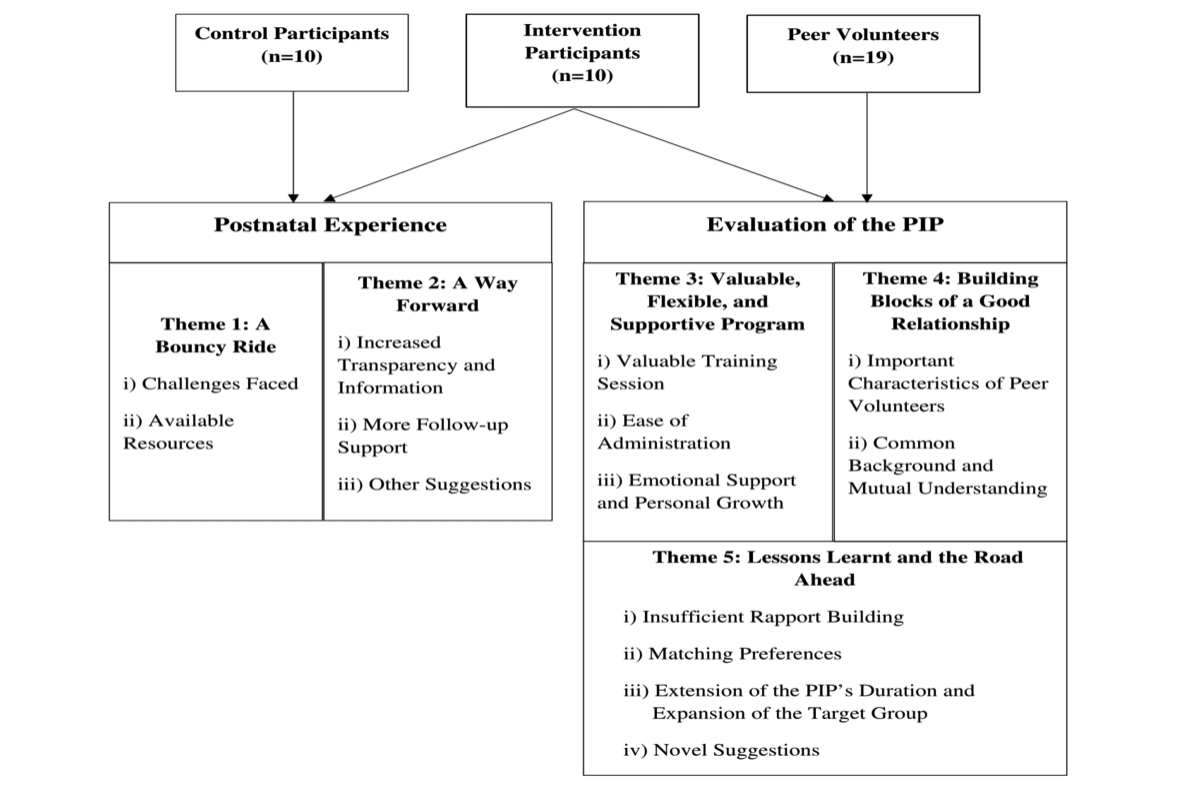
Overview of themes and subthemes. PIP: peer support intervention program.

Two overarching themes comprising five subthemes were generated: postnatal experience (a bouncy ride, a way forward) and evaluation of the PIP (valuable, flexible, and supportive program; building blocks of a good relationship; and lessons learned and the road ahead). A detailed breakdown of the themes and subthemes is shown in Figure 1. Exact quotes from the participants were used to represent their emic views, and their identities were coded to allow for anonymity, with mothers in the control group labeled with C, mothers in the intervention group labeled with T, and peer volunteers labeled with P.

**Table 1:** Characteristics of study participants (control n=10, intervention n=10, volunteers n=19).

Characteristics		Control	Intervention	Peer volunteers
Age (years), mean (SD), range		32.0 (5.0), 25-40	31.5 (4.7), 25-40	40.4 (7.9), 25-54
**Ethnicity, n (%)**				
	Chinese	6 (60)	4 (40)	17 (90)
	Malay	3 (30)	6 (60)	1 (5)
	Indian	1 (10)	0 (0)	1 (5)
**Marital status, n (%)**				
	Married	10 (100)	9 (90)	17 (90)
	Single	0 (0)	1 (10)	2 (11)
University graduates, n (%)		7 (70)	9 (90)	13 (68)
**Monthly household income (S$), n (%)**				
	<3000	3 (30)	1 (10)	3 (16)
	3000-5000	3 (30)	3 (30)	4 (21)
	>5000	4 (40)	6 (60)	9 (47)
Attended antenatal class, n (%)		6 (60)	5 (50)	7 (37)
**Mode of delivery, n (%)**				
	Normal vaginal delivery or water birth	8 (80)	5 (50)	8 (42)
	Instrument delivery	0 (0)	3 (30)	5 (26)
	Cesarean delivery	2 (20)	2 (20)	6 (32)
**Number of children, n (%)**				
	One	8 (80)	8 (80)	5 (26)
	Two	2 (20)	0 (0)	8 (42)
	Three or more	0 (0)	2 (20)	6 (32)
Breastfeeding, n (%)		9 (90)	9 (90)	17 (90)

### Theme 1: A Bouncy Ride

This theme describes the bumpy postnatal experiences of mothers including the challenges of breastfeeding, their physical and emotional states, and the types of support received.

#### Challenges Faced

Mothers from both the intervention and control groups frequently described breastfeeding as stressful and difficult. Heavy infant care responsibilities also left mothers feeling exhausted, tired, and overwhelmed. Receiving support from family members served as a double-edged sword with a few mothers reporting conflicts in parenting styles and opinions due to generation gaps causing additional stress.

Different sides of [the] family have different cultures and different upbringings, so sometimes, it’s like you have too many inputs, you get stressed...

T9

A combination of the above factors resulted in many first-time mothers feeling lost, worried, and scared.

#### Available Resources

During the short hospitalization period, all mothers were satisfied with the support received from health care professionals, often citing them as caring, encouraging, assuring, and patient when guiding them in breastfeeding and answering their queries.

The majority of mothers from both groups were grateful for the high instrumental support provided by their family members, especially their partners. With family members helping to shoulder most household responsibilities and infant care tasks, mothers had more *me time* to catch a breather and rest.

I feel much more relieved because I know the baby is [being] taken care [of] by my husband...It helped me to get back my sleep...

T9

Baby care information was often sought from friends who had recently given birth, otherwise mothers were embarrassed to seek hands-on support from them. Facebook groups, forums, and websites were also commonly referred to as information sources and support, but some mothers remained skeptical of the credibility of online information.

### Theme 2: A Way Forward

In general, mothers indicated a need for more informed decisions and social support to improve their birth experiences.

#### Increased Transparency and Information

Mothers from both groups identified a high need for hospitals to provide more information to facilitate informed decision-making in terms of medical costs and medical procedures and to increase the awareness of postnatal blues and the availability of support sources.

I’m unaware of how this system is like...We didn’t do a lot of research...We thought that they would inform us...

T9

#### More Follow-Up Support

In consideration of the 1-month confinement period adhered to by most mothers, there were mixed preferences for home visits and increasing the frequency of follow-up phone calls by health care professionals.

It’s difficult to tell to explain the situation over the phone...You really have to, like, watch and observe the problem...

C7

There could be...more follow-ups...Actually, a phone call is good enough...At least you know that you’re being taken care of...

T3

Mothers in the control group also expressed a need for peer volunteers as an additional support.

My friends are married...but they don’t have [a] baby, so there’s too little information I can get from them...If there’s a peer volunteer for me, I’ll definitely find it very good...

C10

Other mothers suggested having a breastfeeding buddy or befriender in the hospital to stand in nurses’ place and guide them in breastfeeding techniques.

Some volunteer that could at least help out a bit...rather than have nobody, cause the nurses, they’re busy...I think it will make the journey a bit less stressful...

T4

#### Other Suggestions

Although many mothers referred to the UK-based website babycentre for baby care information, they highly preferred a baby mobile app that could provide more locally relevant information, such as on confinement practices. They also mentioned that they would find the mobile app more reputable and reliable if it were moderated by health care professionals from local hospitals.

Google will always come up with...from [the] United States, [the] UK, that kind...Their advice is very westernized, may not be what Singaporeans follow...especially confinement...

T4

Baby Center was the most popular thing...If there was something with the hospital...we will think it’s more reputable...

C9

Other suggestions included mobile app features such as having baby food recipes, instant messaging with health care professionals, and an online portal with the hospital to check and update baby appointments.

### Theme 3: Valuable, Flexible, and Supportive Program

This theme summarizes the feedback of peer volunteers and mothers who received support (the intervention group only) on the usefulness of the PIP. Overall, the peer volunteers were satisfied with the informative training session. The flexibility of the program was also mentioned by both mothers and peer volunteers, which made them feel supported.

#### Valuable Training Session

Peer volunteers found the training by the psychiatrist very useful, informative, and helpful in setting their expectations. It also enhanced their self-confidence, sensitivity, and awareness toward at-risk mothers.

It kind of gives us the assurance...the confidence level that we can do it...

P8

It was helpful in creating more awareness in me...It helps me to be a bit more sensitive...

P3

A few peer volunteers even mentioned how the training session established a support system for them and created a sense of belonging since they shared a common past and common goal in this study.

The sense of belonging is like...cause we all felt the need to support these mothers...Either it’s because of our own experiences or there’s a sense of same heart in supporting these women...

P16

However, there were also suggestions for more culture-specific information and simulated roleplays to increase preparedness and their abilities to cater to mothers from different cultures.

#### Ease of Administration

Both peer volunteers and mothers who received the PIP praised its flexibility as the mode of contact was to their convenience and preferences, which was mostly through text messages.

It’s very hard for me to talk on the phone because of the baby and my other kid, so WhatsApp is the best mode cause I can reply as and when I am free...

T8

WhatsApp is convenient, like, anytime that they reply, you can just reply at work...Very flexible...You can accommodate both working moms and stay-at-home moms...

P19

Although a few pairings attempted to converse through phone calls, both parties admitted it was hard to match their busy schedules.

The baby is a bit difficult to, you know, find the right time...It’s a bit difficult to arrange...

T1

I don’t want to call without warning...When I try to set a time, they don’t commit, then it’s not very nice also if I just call unannounced...

P4

#### Emotional Support and Personal Growth

The majority of the mothers were appreciative of the extra listening ear and felt they had another friend to talk to and they were not alone. They also felt more comforted and reassured and had reduced negative feelings after receiving continuous peer support.

Whenever I have issues, she will comfort me...that I’m not the only one going through it, and it’s only normal...It was very nice to have somebody additional apart from my own family.

T8

Almost all of the peer volunteers found the PIP experience meaningful, enriching, and fulfilling and felt happy that their previous experiences could benefit other mothers. Some even took it as a chance to self-reflect and for self-improvement.

I can learn something new...I can improve myself...The more I give, then the more I feel happy. I just want to make sure that they didn’t get depression or stressed.

P9

### Theme 4: Building Blocks of a Good Relationship

This theme describes the essential traits of a befriender and the basic underlying foundation of a good relationship.

#### Important Characteristics of Peer Volunteers

Most recipients of the peer support reported that the friendliness, sincerity, proactiveness, positivity, and commitment shown by the peer volunteers allowed them to gradually open up and share their problems.

She took [the] initiative to check on me when I was having my confinement...She was also quite cheerful...After that, she still asked me occasionally how I was...Despite her having so many kids, she still bothered to remember this peer volunteer friend...It was a two-way thing…Quite assuring to know that somebody could help me when I needed [it].

T4

Many peer volunteers shared that being understanding and open-minded was important in establishing a good relationship. Being perceptive was also vital in gauging the responses of mothers and knowing when to not be overimposing. Additionally, a strong mentality and awareness of their role limitations also helped them to cope with the lack of responses and not to take it personally.

I would also remind myself not to take it personally...The whole point of this is to be open-minded and to help them...

P4

#### Common Background and Mutual Understanding

Mothers and peer volunteers who reportedly had closer friendships found it easier to relate with one another due to various common factors (ie, marital status and working background), and they possessed a mutual understanding of each other’s busy schedule and that delayed replies were inevitable.

I’m a single parent...and the lady is also a single parent, so, like, there are some common factors...We kind of support each other...so it’s more like a two-way thing...which is why I say she is more like a friend.

T3

### Theme 5: Lessons Learned and the Road Ahead

#### Insufficient Rapport Building

A few mother-peer volunteer pairs faced initial discomfort in sharing their thoughts with a stranger. The lack of connection resulted in awkwardness and one-sided, superficial relationships.

Sometimes, I don’t feel very connected to the person that I call...so, sometimes, it gets awkward during the phone conversation.

T2

A lack of response from mothers also added stress and uncertainty for some peer volunteers as they were unable to identify the mother’s needs, hence disabling their support efforts. Peer volunteers were also unsure if they had provided adequate help or whether they were disturbing the mothers.

It’s a bit discouraging, so...I know they may not have the time to read the message or reply to me...I can’t do anything...I cannot help much. I don’t know what’s going on.

P2

#### Matching Preferences

In order to increase relevance to self, mothers generally preferred to be matched with a volunteer of similar age, same ethnicity, employment status, marital status, recency of childbirth, and similar ages of children. One mother expressed concern over the age gap with her peer volunteer.

She doesn’t quite understand the current fast pace of a working environment and young parents like us who have to juggle so many things...

T6

However, in order to provide relevant and effective advice, peer volunteers preferred to be matched based on delivery mode, mode of feeding, and socioeconomic and employment status.

#### Extension of the Peer Support Intervention Program’s Duration and Expansion of the Target Group

Both mothers and peer volunteers agreed that the PIP should be extended for at least 2 to 3 months postpartum since the onset of PND is unpredictable.

It will help, especially the second month...when confinement lady is gone...when my husband goes back to work, then you’re left alone with the kids...You need this added support.

T5

Since PND is hard to detect in the early stages, the PIP was highly recommended to be made available to all new mothers as a safety net. Most participants acknowledged that having additional support would benefit all mothers emotionally, regardless of their risks of depression

Some of them, they are strong enough, but sometimes, they just need a listening ear to release their stress...

P15

#### Novel Suggestions

Mothers and peer volunteers were highly in favor of at least one session of face-to-face meet-up, which would allow easier rapport building and let peer volunteers provide more substantial instrumental support.

Maybe one [session] could be like a visit, to see them, say hello...Maybe they will feel better...It will be easier to build the rapport.

P19

Developing a mobile app to locate and contact nearby befrienders who could help during emergencies was also suggested. This would increase convenience, reduce the hassle of pairing peer volunteers, and facilitate the natural occurrence of friendships.

## Discussion

### Principal Findings

Overall, postnatal experiences were highly similar in both groups of mothers, such as common breastfeeding issues and physical fatigue. A large-scale review of the effectiveness of various types of breastfeeding support concluded that regularly scheduled face-to-face support by health care professionals has a higher success rate in teaching mothers to breastfeed [58]. Findings from another local study that used educational parenting videos emphasized mothers’ need for both theory and hands-on learning through visual means [55]. As the PIP did not focus on the physical or breastfeeding needs of mothers, this could be the reason that mothers from the intervention group were equally distressed about their physical and breastfeeding needs.

In terms of emotional experience, mothers who received the PIP had reduced negative feelings and better understanding and acceptability of their emotional situations; hence, they were better able to enjoy their birth experiences. Mothers without additional support felt lost, anxious, and alone, which has been commonly reported in other studies [55-60]. The perceived effectiveness of the PIP in providing adequate emotional support for mothers is also supported by the study findings of Dennis et al [46], in which mothers benefitted from the emotional support received from peer volunteers.

Although mothers in both groups were highly satisfied with the quality and competency of health care providers during their hospitalization stays, they were disappointed with the lack of transparency and insufficient information from health care professionals, which hindered their informed decision making processes. Being involved and making well-informed child care decisions gives mothers a sense of empowerment, responsibility, and preparedness, which promotes better health outcomes and healthier physical and psychological well-being and even affects the long-term health and well-being of their children [61]. Mothers also voiced the need for more hospital follow-ups and continuity of care in terms of phone calls and home visits. Studies have shown that continuity of care is often lacking due to resource constraints, such as a lack of manpower [62], but patients who received continuity after hospital discharge had better health care outcomes, higher satisfaction rates, and more cost-effective health care [59,63]. Therefore, in order to promote positive postpartum experiences, a multidimensional approach to improve current hospital care in the form of follow-up multimodal educational programs needs to be considered.

Interestingly, when describing the support received from family and their partners, mothers from both groups only mentioned their satisfaction with the instrumental support received from their parents, in-laws, and partners. This is supported in a study by Chen and colleagues [64] that revealed that Asians tend to provide more problem-focused support than European Americans who tend to provide more emotion-focused support. This is mainly due to the conservative, collectivist Asian culture whereby indirect support, which avoids mention of the stressor, is preferred and includes displays of attentiveness and companionship from their loved ones [22]. However, conflicts with in-laws in terms of parenting styles and differences in opinions due to generation gaps were commonly reported, adding emotional stress for mothers. As respect for elders is highly observed in the Asian culture, new mothers often have to suppress their emotions, and having a poor relationship with one’s mother-in-law potentially jeopardizes her marital relationship and increases risks of PND [11,26,27,65]. Chen and colleagues [64] concluded that the most effective type of social support is a delicate balance between autonomy and dependence. Considering that negative childbirth experiences can cause partner and family strain, an external support in the form of peer-volunteers might be more ideal when providing a nonjudgmental listening ear [66].

### Peer Support Intervention Program

Similar to other peer support studies [47,67,68], our peer volunteers underwent mandatory training to learn adequate support provision for at-risk mothers, which most found useful in setting personal expectations and gaining confidence and preparedness. Additionally, since peer volunteers were mothers with a history of PND, training sessions also served as a platform for mothers with similar pasts to connect and form a community of support [47].

In an effort to increase the accessibility and flexibility of support with minimal disruption to the mothers’ lives, communication through technology-based platforms was adopted. This aspect was praised by both recipients and peer volunteers as correspondence was flexible and suited to their own levels of comfort, whether it was through text messages or phone calls. Due to unpredictability and difficulty in arranging fixed calling times, most mothers and volunteers preferred correspondence through text messages. However, a few reported the lack of a personal touch and difficulty in gauging mothers’ responses over text messages, with some mothers also suggesting at least one home visit or face-to-face meet-up. A review by Shaw [60] acknowledged the effectiveness of peer-support in reducing PND, but maternal satisfaction was higher for home visits. Videoconferencing was not used in this study, but it has been shown to improve mental health outcomes [69] and increase confidence and was deemed to be almost equivalent to the physical presence of a peer volunteer [43]. Parents in a study by Linberg [43] also felt that the midwife understood their situations better with added nonverbal communication through videoconferencing. Hence, the option of using videoconferencing can be considered in future local studies to support mothers at risk of PND.

Initiating a socially meaningful relationship of trust involves certain key characteristics of peer volunteers: positivity, determination, openness, proactiveness, sincerity, and respect. These qualities are critical to successful program delivery and enable peer volunteers to overcome challenges and stress and establish effective functional relationships [68]. Mothers and volunteers shared that the mutual understanding and identification that drew on the shared experiences of parenthood and social circumstances increased feelings of connectedness and ease of sharing. Despite the differences in personality, the sharing of experiences with someone of similar backgrounds, attitudes, and experiences facilitates trust-building and easier communication [68,70]. The matching of peer volunteers to mothers is an important component of successful peer-mentoring as the provision of support should be targeted to fit the nature and situation of the family [46,50,71].

This PIP was also seen as a two-way support system. While most mothers were appreciative of the extra listening ear and support provided that improved their overall emotional well-being, peer volunteers were also glad for the opportunity to impact other lives with their experiences and felt happier after volunteering. Expressing satisfaction with their roles, many volunteers felt empowered and took this chance to learn new things to enhance their personal growth and self-discovery. Similarly, in other studies peer volunteers felt emotionally rewarded and enriched by their interpersonal relationships; given the chance, they would volunteer again [47,66].

However, challenges and stressful moments arose for some peer volunteers when communication was only one-sided. Mothers and volunteers attributed it to insufficient rapport-building and the discomfort of sharing with a stranger. A few volunteers perceived some relationships as superficial and obligatory, while some mothers felt that they did not need the additional support. This is common in the Asian culture as the stigma of mental illness is related to the loss of face beyond the individual level [72,73]. In a culture that emphasizes emotional restraint, avoidance of shame, and saving face, it may take a longer time for mothers to warm up and share their problems with volunteers [74].

### Implication for Future Research and Clinical Practice

Given the easy accessibility and wide availability of technological devices today, technology-based peer support is a potentially time-saving and cost-effective way to extend social support to mothers in need. Being able to provide mothers with a personalized continuity of care post–hospital discharge should incentivize health care sectors to dedicate more resources to train peer volunteers and implement the technology-based peer support to a wider population.

In consideration of the collectivistic culture and strong interdependence on family, future studies on technology-based peer support in Asia can involve partners and family members, which might reduce the emotional strain between mothers and family members during the stressful postpartum period. Future research can also consider including videoconferencing or developing a peer volunteer mobile app with detailed profiles to provide more targeted forms of support. Given that breastfeeding remains a key stressor to mothers, future interventions can adopt a holistic approach of providing both instrumental and emotional support to new mothers. Additionally, the introduction of peer volunteers to mothers should ideally take place during the third trimester, when mothers still have the time to build rapport. Last, the practice of the confinement period may see mothers receiving more help from family, in-laws, or confinement nannies during the immediate postpartum period for one month [24]. Instead, the second month postpartum, when all added help is depleted, may be more crucial as mothers resume work and the full responsibility of infant care. Therefore, the extension of the PIP’s duration will be beneficial to mothers during this period.

### Strengths and Limitations

Unique to this study is the valuable insight of cultural influences on preferred types of social support and the help-seeking behaviors of Asian mothers in the postpartum period. The inclusion of both mothers’ and peer volunteers’ perspectives also provided a well-rounded overview of the PIP. To our knowledge, this is the first technology-based peer support intervention in Asia. Therefore, the study findings are vital in informing future research on PND prevention using technology-based peer support, especially in an Asian context.

However, the cross-sectional nature of this study disallows the comparison of the pretest and posttest perceptions of mothers, making it difficult to determine the effectiveness of the intervention. Additionally, due to previous childbirth experiences, the sample inclusion of non–first-time mothers may bias the findings positively and provide inaccurate information on the usefulness of the PIP. Hence, future interviews should consider interviewing an equal number of first-time and experienced mothers and analyzing if there is any uniqueness in their individual experiences of receiving the PIP.

### Conclusion

This study not only provided insight into the postnatal experiences and needs of at-risk Asian mothers but also evaluated the perceptions of both recipients and administrators of the technology-based PIP. The positive endorsement by mothers and volunteers suggests the PIP’s success and usefulness in meeting mothers’ postpartum needs. The technology-based PIP has the potential to connect with and give support to vulnerable mothers and enable them to access services in ways that complement the work of health professionals. However, given the suggestions for improvement by stakeholders, more research and rigorous testing are needed to further refine this approach before its implementation to a wider community.

## Appendix

Multimedia Appendix 1 Semistructured interview guide for participants and peer volunteers.

Multimedia Appendix 2 Consolidated Criteria for Reporting Qualitative Research (COREQ) checklist.

## Abbreviations

PIP: peer support intervention program
PND: postnatal depression
RCT: randomized controlled trial
