# Supplementary material for: Evaluation of a Technology-Based Peer-Support Intervention Program for Preventing Postnatal Depression (Part 2): Qualitative Study
Source: J Med Internet Res. 2019 Aug 29;21(8):e12915. doi: 10.2196/12915 (PMC6740164; doi:10.2196/12915)
Supplement: Multimedia Appendix 1 [file jmir_v21i8e12915_app1.docx]

| **No.** | **Probing Questions (Control group)** |
| --- | --- |
|  | How do you find the postnatal care provided to you by the hospital? |
|  | What are the strengths and weaknesses of the postnatal care provided by the hospital? |
|  | Do you have any suggestion for the improvement of the postnatal care provided to you? |
|  | How do you feel about the current support you have? |
|  | Do you think that you should be receiving additional support during this period? |
|  | What kind of support would you prefer to receive? What would you like to see in your preferred means of support? |

| **No.** | **Probing Questions (Intervention group)** |
| --- | --- |
|  | Were you satisfied with the peer support intervention program? Which areas? |
|  | Was the peer support intervention program provided to you useful in improving your emotional wellbeing? If ‘yes’, how? If ‘no’, why not? |
|  | What were the strengths and weaknesses of the peer support intervention program? |
|  | Do you think it is worthwhile to spend extra time receiving this Peer-support Intervention Program intervention? Any suggestions for improvement? |
|  | How was the quality of your relationship with the peer volunteer? |
|  | Do you think the peer volunteer was helpful in providing advice and support? In what way? |
|  | Do you think your assigned peer volunteer was a good match? Do you have any preferred pairing system or any factors that we should take into consideration? |
|  | How do you find the postnatal care provided to you by the hospital? |
|  | Do you have any suggestions for the improvement of postnatal support provided to you? |

| **No.** | **Probing Questions (Peer volunteers)** |
| --- | --- |
|  | How did you find the training given to you? Was it good? In what way? If ‘not so good’, why? |
|  | How did you find the delivery of this intervention? |
|  | Did you face any difficulties? If ‘yes’, can you give an example? How did you overcome it? |
|  | How were your interactions with the mothers? |
|  | How did you find your role as a volunteer? |
|  | Did volunteering and participation in this study have any effects on you? Positive/negative? In what way? |
|  | Do you think it is beneficial to support mothers by laypersons like yourself in the future? Why? |
|  | What were the strengths and weaknesses of this intervention program? |
|  | Do you have any suggestions for the improvement of this intervention? |
